# Targeting autophagy to enhance chemotherapy and immunotherapy in oral cancer

**DOI:** 10.3389/fimmu.2024.1535649

**Published:** 2025-01-07

**Authors:** Xiaoli Zeng, Yue Chen, Jing Wang, Miao He, Junyao Qiu, Yun Huang

**Affiliations:** ^1^ Department of Oncology, The First Affiliated Hospital of Gannan Medical University, Ganzhou, Jiangxi, China; ^2^ Jiangxi "Flagship" Oncology Department of Synergy for Chinese and Western Medicine, The First Affiliated Hospital of Gannan Medical University, Ganzhou, Jiangxi, China; ^3^ Department of Oncology, Jiangxi Clinical Medical Research Center for Cancer, The First Affiliated Hospital of Gannan Medical University, Ganzhou, Jiangxi, China; ^4^ Department of Gastroenterology, The Affiliated Ganzhou Hospital of Nanchang University, Ganzhou, Jiangxi, China; ^5^ Department of Otolaryngology, The Affiliated Ganzhou Hospital of Nanchang University, Ganzhou, Jiangxi, China

**Keywords:** autophagy, oral cancer, apoptosis, chemotherapy, radiotherapy, immunotherapy

## Abstract

Oral cancer is a highly malignant disease characterized by recurrence, metastasis, and poor prognosis. Autophagy, a catabolic process induced under stress conditions, has been shown to play a dual role in oral cancer development and therapy. Recent studies have identified that autophagy activation in oral epithelial cells suppresses cancer cell survival by inhibiting key pathways such as the mammalian target of rapamycin (mTOR) and mitogen-activated protein kinase (MAPK), while activating the adenosine monophosphate-activated protein kinase (AMPK) pathway. Inducing autophagy promotes degradation of eukaryotic initiation factor 4E, thus reducing metastasis and enhancing the efficacy of chemotherapy, radiotherapy, and immunotherapy. Furthermore, autophagy induction can modulate the tumor immune microenvironment and enhance antitumor immunity. This review comprehensively summarizes the relationship between autophagy and oral cancer, focusing on its mechanisms and therapeutic potential when combined with conventional treatments. While promising, the precise mechanisms and clinical applications of autophagy inducers in oral cancer therapy remain to be elucidated, offering new directions for future research to improve treatment outcomes and reduce recurrence.

## 1 Introduction

Oral cancer is one of the most prevalent cancers of the head and neck region, commonly affecting the lips, buccal mucosa, and tongue. Its high malignancy, recurrence, and metastasis significantly impair patients’ quality of life and impose substantial economic burdens. Key risk factors include alcohol consumption, tobacco use, betel quid chewing, and human papillomavirus (HPV) infection. Despite advancements in surgical, radiotherapeutic, and chemotherapeutic strategies, the overall 5-year survival rate of oral cancer patients remains approximately 50%. However, early detection can improve survival rates to nearly 80%, highlighting the urgent need for innovative therapeutic approaches (1).

Autophagy, a highly conserved cellular process, plays a critical role in maintaining cellular homeostasis and responding to stressors such as hypoxia, nutrient deprivation, and DNA damage (2). It facilitates the degradation and recycling of damaged organelles, misfolded proteins, and other cytoplasmic components. Dysregulation of autophagy has been implicated in cancer, where it exerts dual roles as a tumor suppressor in early stages and as a tumor promoter in advanced cancers (3).

In oral cancer, autophagy regulates a range of processes, including carcinogenesis, metastasis, and response to therapy. Recent studies have identified potential therapeutic applications of autophagy modulation, particularly in enhancing the efficacy of chemotherapy, radiotherapy, and immunotherapy. Although autophagy inducers show potential in enhancing therapeutic effects, their potential side effects and safety concerns in clinical applications need to be carefully considered in treatment strategies. This review aims to elucidate the relationship between autophagy and oral cancer progression, discuss the underlying molecular mechanisms, and explore its potential as a therapeutic target.

## 2 Autophagy and oral cancer progression

### 2.1 Role of autophagy in oncogenesis

Autophagy has emerged as a critical regulator in the progression of oral cancer, influencing both the initiation and advancement of the disease. Understanding its dual roles in tumor suppression and promotion provides valuable insights into its potential as a therapeutic target. Autophagy maintains genomic stability in normal oral epithelial cells by removing damaged DNA and organelles (4). Dysregulation of autophagy-related genes (ATGs), such as ATG5 and ATG7, leads to oxidative stress, the accumulation of dysfunctional proteins, and genomic instability, promoting tumorigenesis. For example, loss of ATG5 in oral epithelial cells has been associated with increased reactive oxygen species (ROS) production, contributing to DNA damage and malignant transformation (5). The tumor suppressor gene TP53, which encodes P53, is a pivotal regulator of autophagy. Under conditions of stress, such as nutrient deprivation or hypoxia, P53 activates autophagy by inhibiting the mTOR pathway and modulating the Bcl2/beclin-1 complex (6). This process prevents DNA damage and delays carcinogenesis. However, mutations in TP53, which occur in up to 80% of oral squamous cell carcinoma (OSCC) cases, impair autophagy activation, promoting tumor progression (6). Liu et al. (7) demonstrated that in a hamster oral cancer model, autophagy activation in precancerous lesions was associated with lower levels of DNA damage markers, suggesting a protective role in early carcinogenesis. Conversely, reduced autophagy activity in malignant lesions correlated with increased DNA damage, highlighting the importance of autophagy in preventing tumor initiation.

In the early stages of oral cancer, autophagy primarily functions as a tumor suppressor. It maintains genomic stability by facilitating the clearance of damaged organelles and misfolded proteins, which prevents oxidative stress and DNA damage. Studies have shown that autophagy activation in precancerous lesions leads to lower levels of DNA damage markers and protects against malignant transformation (5–7). In these early stages, autophagy acts as a defense mechanism, delaying carcinogenesis. However, in advanced stages of oral cancer, autophagy may contribute to tumor progression by promoting cancer cell survival under adverse conditions such as nutrient deprivation, hypoxia, and chemotherapy. It helps maintain cellular homeostasis by degrading cellular components, enabling tumor cells to resist apoptosis and promoting metastasis (8–10). Additionally, autophagy in advanced tumors has been shown to enhance the epithelial-mesenchymal transition (EMT) process and facilitate immune evasion, further driving cancer progression (8, 9).

### 2.2 Impact of autophagy on tumor metastasis

Metastasis, primarily involving cervical lymph nodes in oral cancer, is a major cause of treatment failure and mortality (11). Autophagy plays a complex role in modulating tumor metastasis by influencing EMT, matrix remodeling, and invasion (8) MTA2, a key driver of tumor metastasis, has been shown to suppress autophagy-related protein LC3-II expression in oral cancer cells, facilitating metastasis. Silencing MTA2 using shRNA restored autophagy and inhibited tumor migration and invasion. Autophagy also regulates EMT, a process critical for cancer cell migration and invasion (9). Liang et al. (10) reported that in rapamycin-treated OSCC cells, high expression of METTL14 enhanced autophagy, inhibited EMT, and suppressed metastatic potential. METTL14-mediated autophagy degraded eIF4E, reducing cancer cell invasion. Additionally, Autophagy significantly impacts the tumor microenvironment (TME) by modulating immune cell infiltration, cytokine secretion, and extracellular matrix (ECM) remodeling (12). It enhances antigen presentation by facilitating the processing and presentation of tumor antigens on major histocompatibility complex (MHC) molecules, thereby improving T cell activation and antitumor immune responses (13). Additionally, autophagy maintains the homeostasis and effector functions of T cells, NK cells, and macrophages, which are crucial for effective immune surveillance and response against tumor cells (14). Autophagy influences the secretion of cytokines and chemokines that modulate immune cell recruitment and activity within the TME. For instance, autophagy enhances the secretion of CXCL10, a chemokine that attracts effector T cells and NK cells to the tumor site, thereby strengthening the immune response (15). Furthermore, autophagy regulates the production of pro-inflammatory cytokines that contribute to a more favorable antitumor environment. Autophagy affects ECM remodeling by regulating the degradation of ECM components and the activity of MMPs. This modulation of the ECM can influence cancer cell migration and invasion, thereby affecting tumor progression and metastatic dissemination. By altering the ECM, autophagy can create a microenvironment that either supports or inhibits tumor growth and spread (16).

## 3 Molecular mechanisms underlying autophagy regulation

**Figure 1:**
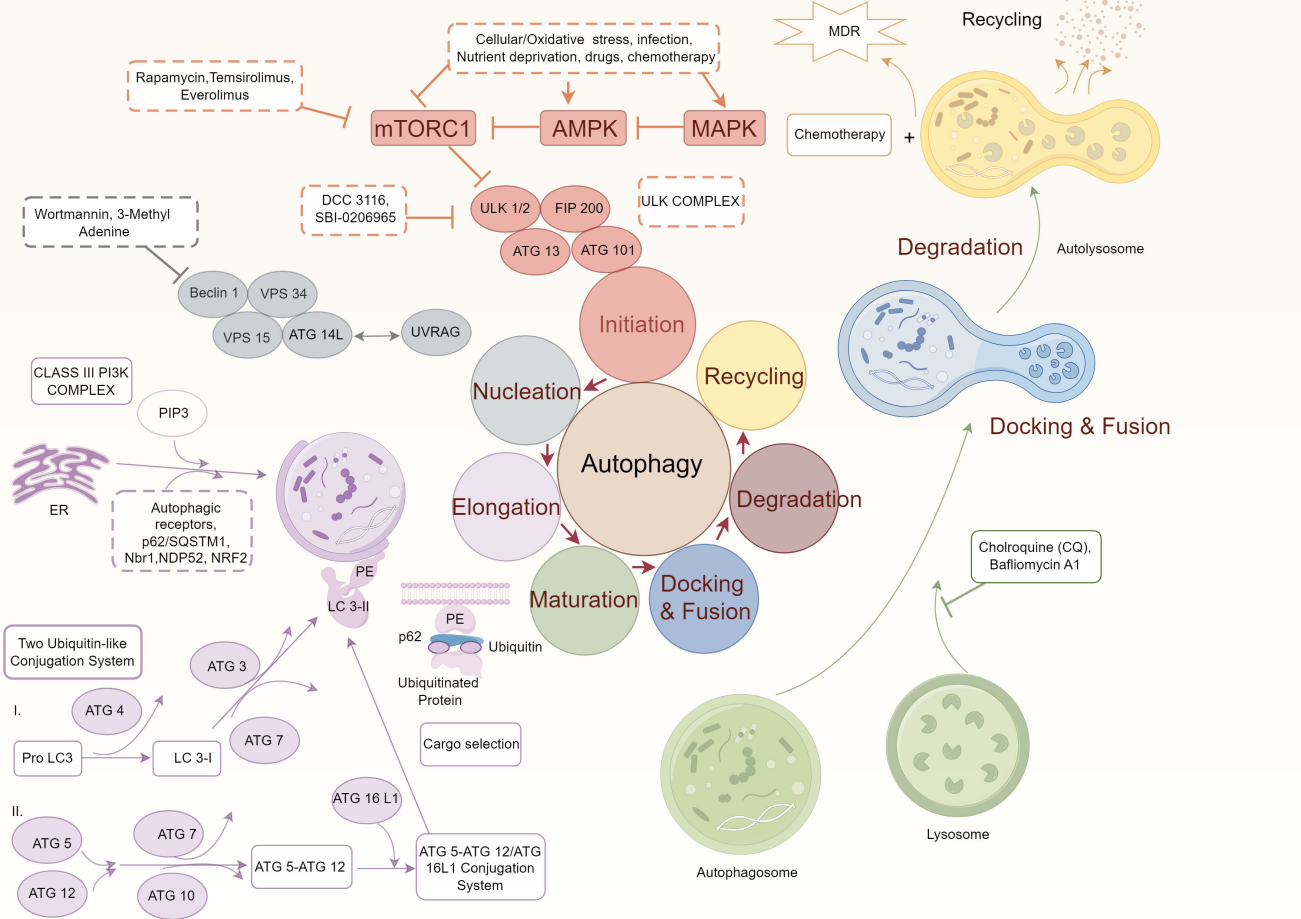
Molecular mechanisms underlying autophagy regulation.

Autophagy is a tightly regulated process that plays a critical role in maintaining cellular homeostasis and responding to various stressors. In OSCC, several signaling pathways influence the regulation of autophagy, dictating its dual roles in tumor suppression and progression. This section explores three key pathways—mTOR, AMPK, and Beclin-1/Bcl2 interactions—and their therapeutic implications in oral cancer (**Figure 1**).

### 3.1 mTOR pathway

The mechanistic target of rapamycin (mTOR) is a serine/threonine kinase and a central regulator of autophagy. It integrates signals from nutrients, growth factors, and cellular energy levels to suppress autophagy under favorable conditions (17–20). In OSCC, mTOR is frequently hyperactivated due to mutations or dysregulation of upstream signaling pathways, leading to autophagy inhibition and promoting uncontrolled tumor growth. mTOR inhibits autophagy by ULK1 at Ser757, disrupting its interaction with AMPK and preventing autophagosome formation (21). Pharmacological inhibition of mTOR, using drugs such as rapamycin and its analogs, restores autophagic activity. Rapamycin binds to FKBP12, forming a complex that specifically inhibits mTORC1, thereby inducing autophagy. Additionally, natural compounds like curcumin inhibit the mTOR pathway by downregulating upstream activators such as PI3K/Akt, further promoting autophagy. Pre-clinical studies in OSCC have demonstrated that rapamycin enhances autophagosome formation, reduces cancer cell proliferation, and increases the sensitivity of cancer cells to chemotherapeutic agents (22–24). Additionally, mTOR inhibitors improve the tumor microenvironment by increasing immune cell infiltration, further enhancing antitumor immune responses.

Natural compounds targeting the mTOR pathway have also shown promise in inducing autophagy in OSCC. For instance, curcumin and its derivative MTH-3 inhibit the EGFR/AKT/mTOR signaling pathway, upregulating autophagy-related markers such as LC3B-II and p62 while downregulating p38 MAPK expression, thereby reducing OSCC cell viability (25). The molecular mechanism of curcumin involves the inhibition of the PI3K/Akt pathway, leading to decreased phosphorylation of mTOR and its downstream targets, which promotes autophagosome formation. Pterostilbene restores autophagic activity in cisplatin-resistant OSCC cells by activating autophagy-related genes, including ATG5, ATG7, and Beclin-1, through modulation of the AKT pathway, leading to enhanced cancer cell death. Similarly, pterostilbene restores autophagic activity in cisplatin-resistant OSCC cells by activating autophagy-related genes, including ATG5, ATG7, and Beclin-1, through modulation of the AKT pathway, leading to enhanced cancer cell death. Potential antitumor drugs like chlorpromazine have also been found to induce autophagy in gingival cancer cells by upregulating autophagy-related proteins (Atg5, Atg7, Atg12, Beclin-1, and LC3A/B-II) while downregulating negative regulators (mTOR, PI3K, Akt, and p70S6K) (26–28). This mechanism promotes apoptosis and suppresses cancer cell growth. Other compounds, such as ursolic acid, enhance autophagy by modulating multiple pathways, including mTOR, AKT, and p38 MAPK, while inducing apoptotic cell death in OSCC cells.

### 3.2 AMPK pathway

AMPK is a cellular energy sensor that responds to low ATP levels by promoting catabolic pathways, including autophagy, to restore energy homeostasis. AMPK activates autophagy through two primary mechanisms (29). Direct Activation of ULK1: AMPK phosphorylates ULK1 at Ser317 and Ser777, directly stimulating its kinase activity and initiating autophagosome formation. This phosphorylation occurs at a different site than mTOR, enabling AMPK to bypass mTOR inhibition under energy-deprived conditions. Inhibition of mTOR: AMPK phosphorylates TSC2 and Raptor, components of the mTORC1 complex, leading to mTOR inhibition (30). This dual action ensures robust activation of autophagy even in the presence of conflicting upstream signals.

In oral cancer, AMPK agonists like metformin and AICAR have shown promise in preclinical studies. Metformin induces autophagy by activating AMPK and reducing cancer cell viability. In OSCC xenograft models, metformin-mediated autophagy has been associated with decreased tumor growth and enhanced sensitivity to cisplatin (31). Moreover, AMPK activation modulates metabolic reprogramming in cancer cells, reducing glycolytic flux and oxidative stress, which indirectly promotes autophagic cell death (32).

### 3.3 Beclin-1/Bcl2 interaction

Beclin-1 is a central regulator of autophagy that forms complexes with VPS34 to initiate autophagosome formation (33). However, its activity is negatively regulated by anti-apoptotic Bcl2 family proteins, including Bcl2 and Bcl-XL, which bind to the Bcl2 homology 3 (BH3) domain of Beclin-1, preventing its interaction with VPS34 (34).

In OSCC, overexpression of Bcl2 and Bcl-XL leads to reduced autophagic activity and increased resistance to apoptosis. Disrupting the Beclin-1/Bcl2 complex using BH3 mimetics, such as ABT-737, restores autophagic activity and induces cancer cell death (35). Additionally, small molecule inhibitors that disrupt the Beclin-1/Bcl2 interaction have been shown to effectively induce autophagy. These inhibitors bind to the BH3 domain of Bcl2, preventing its interaction with Beclin-1 and thereby freeing Beclin-1 to initiate autophagy. This strategy not only promotes autophagy but also sensitizes cancer cells to apoptosis, offering a dual therapeutic benefit. Studies have demonstrated that reactivation of Beclin-1 in OSCC cells significantly reduces tumor proliferation and migration (31). Additionally, Beclin-1-mediated autophagy sensitizes OSCC cells to radiotherapy by increasing DNA damage and impairing repair mechanisms.

### 3.4 Autophagy in enhancing chemotherapy and radiotherapy for oral cancer

Autophagy enhances the efficacy of chemotherapy and radiotherapy in oral cancer by modulating signaling pathways and overcoming resistance (36). Curcumin and MTH-3 regulate transcription factor EB and the EGFR/AKT/mTOR pathway, increasing LC3B-II and p62 levels while reducing P38, thereby inducing autophagy and inhibiting OSCC cell activity (37). Pterostilbene promotes autophagy and cell death in cisplatin-resistant OSCC by activating Atg5, Atg7, Atg12, Beclin-1, and LC3-II via the AKT pathway (27). Chlorpromazine increases autophagy-related proteins and inhibits mTOR, PI3K, Akt, and p70S6K, inducing autophagy and apoptosis in gingival cancer cells (38). Furthermore, AMPK activators enhance LC3 and p62 accumulation, inducing apoptosis in SCC2095 cells (39). Ursolic acid modulates AKT/mTOR, NF-κB, ERK, and P38 MAPK, regulating LC3B-II and p62 and enhancing autophagy and apoptosis in OSCC (40).

Combining autophagy inducers with radiotherapy or chemotherapy also shows synergistic effects (18, 41, 42). Tanshinone IIA enhances radiosensitivity by upregulating Beclin-1, ATG5, and LC3-II in SCC090 cells (43). Cordycepin plus radiotherapy elevates ATG5, BECN1, LC3-II, and p62, inducing autophagy, G2/M arrest, and cell death in tongue squamous cell carcinoma (44). Saikosaponin increases radiosensitivity and apoptosis via autophagosome accumulation (30). Rapamycin facilitates autophagosome formation, enhancing radiosensitivity in OSCC (45). USP14 inhibition increases autophagy-related proteins, reducing radio-resistance (46). In chemotherapy, Paris saponin G promotes autophagy through ERK and JNK, increasing Beclin-1, LC3-II, and lowering p62, thereby enhancing chemotherapeutic efficacy (47). Overall, autophagy-induced type II programmed cell death has shown promise across various cancers, including glioblastoma, endometrial, lung, and breast cancers (48, 49). In OSCC, activating AMPK or inhibiting MAPK and mTOR, combined with autophagy inducers, may further improve treatment outcomes and reduce proliferation and metastasis.

## 4 Autophagy and immunotherapy in oral cancer

### 4.1 The relationship between autophagy and tumor immunity

Autophagy plays a dual role in tumor immunity, functioning as both a facilitator of immune surveillance and an enabler of immune evasion (50–53). It significantly influences tumor immunoregulation by modulating antigen presentation, immune cell activity, and the tumor microenvironment. Autophagy is crucial for antigen presentation, particularly in delivering tumor antigens to major histocompatibility complex (MHC) class I and II molecules (54). By promoting the degradation and processing of tumor antigens within antigen-presenting cells (APCs) such as dendritic cells and macrophages, autophagy enhances cross-epitope presentation and subsequently improves T cell activation. Studies have demonstrated that autophagy in tumor cells increases MHC class I-dependent cross-presentation, enhancing CD8+ cytotoxic T lymphocyte-mediated tumor cell killing (55). Similarly, autophagy-mediated MHC class II antigen presentation activates CD4+ helper T cells, contributing to sustained antitumor immune responses (56).

Autophagy also impacts immune cell homeostasis and function within the tumor microenvironment (54). In T cells, autophagy is critical for maintaining metabolic balance and effector function. The loss of autophagy-related gene ATG5 in T cells leads to metabolic dysfunction and impaired proliferation, weakening antitumor immunity (57). Additionally, autophagy clears damaged mitochondria and oxidative stress products, protecting T cells from damage and preserving their effector capabilities. In NK cells, autophagy enhances tumor infiltration and direct cytotoxicity (58). In macrophages (59), autophagy regulates polarization, influencing the immune microenvironment’s pro-tumor (M2) or antitumor (M1) characteristics. By promoting M1 polarization, autophagy may enhance macrophage-mediated antitumor activity (60).

### 4.2 Autophagy and immune evasion

Tumor cells exploit autophagy to evade immune surveillance through various mechanisms (61, 62). Autophagy suppresses apoptosis and the release of antitumor immune factors, such as interferon-γ and tumor necrosis factor-α, allowing tumor cells to evade immune detection (63) Moreover, autophagy regulates immune checkpoint molecules, such as PD-L1, on the tumor cell surface, weakening T cell-mediated immune responses. For instance, autophagy inducers can degrade PD-L1 protein, reversing immune suppression and enhancing antitumor immunity (64). The tumor microenvironment plays a crucial role in determining immune therapy efficacy. Autophagy influences immune cell infiltration and activity by regulating key components of the tumor microenvironment, including cytokines, stromal cells, and angiogenesis. Studies have shown that autophagy enhances the secretion of chemokines, such as CXCL10, by tumor cells, attracting effector T cells and NK cells to tumor sites (65). Additionally, autophagy regulates tumor metabolism, such as lactate and glucose consumption, improving the acidic microenvironment and indirectly boosting immune cell functionality.

### 4.3 Synergistic effects of autophagy and immunotherapy

During cancer progression, tumor cells often escape immune surveillance, contributing to their survival. Cancer immunotherapy aims to enhance various immune response steps to eliminate tumor cells effectively. Autophagy contributes to immunotherapy by enhancing antigen presentation, maintaining lymphocyte homeostasis, promoting T cell activation, and facilitating NK cell infiltration into tumor tissues (66, 67). Autophagy inducers, such as spermidine and trehalose, have shown potential in boosting immune function (68). Current strategies for activating tumor immunity often involve blocking immune checkpoints, such as PD-1/PD-L1. Studies indicate that autophagy induction can reduce PD-L1 expression, promoting tumor cell death (69). For example, anti-PD-1 therapy activates autophagy and enhances tumor cell death in lung cancer (70). In OSCC, combining anti-PD-1 therapy with autophagy inducers, such as rapamycin or metformin, improves antitumor efficacy, prolongs survival, and strengthens immune responses in preclinical models (13). Furthermore, autophagy plays a promising role in tumor vaccine development by enhancing antigen presentation and T cell activation, thereby improving vaccine-mediated antitumor effects (14).

## 5 Conclusion

Autophagy plays a critical role in the pathogenesis and progression of oral cancer. Numerous studies have revealed aberrant expression and dysregulation of autophagy-related genes in oral cancer tissues. In the early stages of oral cancer, inducing autophagy in hyperplastic epithelial cells can inhibit tumor progression. Moreover, promoting autophagy in oral cancer cells has been shown to suppress metastasis and enhance the efficacy of chemotherapy, radiotherapy, and immunotherapy.

However, the dual role of autophagy in tumor promotion and suppression remains a topic of debate. To ensure the safety and efficacy of autophagy inducers in clinical applications, it is essential to thoroughly investigate their potential side effects and safety concerns. The specific targets and optimal timing for autophagy induction in oral cancer therapy require further investigation through comprehensive basic and clinical research. Additionally, the development of targeted drug delivery systems and monitoring biomarkers are key strategies for managing the risks associated with autophagy inducers. It is also essential to consider that tumor cell death is not solely a direct result of drug action but may also stem from changes in the tumor microenvironment. Clarifying the precise mechanisms by which autophagy modulation enhances the synergistic effects of chemotherapy, radiotherapy, and immunotherapy in oral cancer will pave the way for innovative therapeutic strategies. Precise induction of autophagy offers a promising avenue to improve treatment outcomes and reduce the recurrence of oral cancer, potentially representing a significant breakthrough in the field.
